# Testicular Differentiation Occurs in Absence of R-spondin1 and Sox9 in Mouse Sex Reversals

**DOI:** 10.1371/journal.pgen.1003170

**Published:** 2012-12-27

**Authors:** Rowena Lavery, Anne-Amandine Chassot, Eva Pauper, Elodie P. Gregoire, Muriel Klopfenstein, Dirk G. de Rooij, Manuel Mark, Andreas Schedl, Norbert B. Ghyselinck, Marie-Christine Chaboissier

**Affiliations:** 1 University of Nice–Sophia Antipolis, UFR Sciences, Nice, France; 2 INSERM U1091, CNRS UMR7277, iBV, Nice, France; 3 Department of Development and Stem Cells, Institut de Génétique et de Biologie Moleculaire et Cellulaire (IGBMC), CNRS UMR7104–INSERM U964, Illkirch, France; 4 Center for Reproductive Medicine, Academic Medical Center, Amsterdam, The Netherlands; Massachusetts General Hospital, Howard Hughes Medical Institute, United States of America

## Abstract

In mammals, male sex determination is governed by SRY-dependent activation of *Sox9*, whereas female development involves R-spondin1 (RSPO1), an activator of the WNT/beta-catenin signaling pathway. Genetic analyses in mice have demonstrated *Sry* and *Sox9* to be both required and sufficient to induce testicular development. These genes are therefore considered as master regulators of the male pathway. Indeed, female-to-male sex reversal in XX *Rspo1* mutant mice correlates with *Sox9* expression, suggesting that this transcription factor induces testicular differentiation in pathological conditions. Unexpectedly, here we show that testicular differentiation can occur in XX mutants lacking both *Rspo1* and *Sox9* (referred to as XX *Rspo1^KO^Sox9^cKO^* ^)^, indicating that *Sry* and *Sox9* are dispensable to induce female-to-male sex reversal. Molecular analyses show expression of both *Sox8* and *Sox10*, suggesting that activation of *Sox* genes other than *Sox9* can induce male differentiation in *Rspo1^KO^Sox9^cKO^* mice. Moreover, since testis development occurs in XY *Rspo1^KO^Sox9^cKO^* mice, our data show that *Rspo1* is the main effector for male-to-female sex reversal in XY *Sox9^cKO^* mice. Thus, *Rspo1* is an essential activator of ovarian development not only in normal situations, but also in sex reversal situations. Taken together these data demonstrate that both male and female sex differentiation is induced by distinct, active, genetic pathways. The dogma that considers female differentiation as a default pathway therefore needs to be definitively revised.

## Author Summary

Mammalian sex determination is controlled by the paternal transmission of the Y-linked gene, *SRY*. Using mouse models, it has been shown that the main, if not the only, role of *Sry* is to activate the transcription factor *Sox9*, and these two genes are necessary and sufficient to allow male development. Indeed, defects in *Sry* and/or *Sox9* expression result in male-to-female sex reversal of XY individuals. In XX individuals, *Rspo1* is important for ovarian development as evidenced by female-to-male sex reversal of XX *Rspo1* mutants. Since testicular differentiation appears concomitantly with *Sox9* expression, it was assumed that *Sox9* is the inducer of testicular differentiation in XX *Rspo1* mutants. Our genetic study shows that i) neither *Sry* nor *Sox9* are required for female-to-male sex reversals; ii) other masculinizing factors like *Sox8* and *Sox10* are activated in sex reversal conditions; iii) *Rspo1* is the main effector of male-to-female sex reversal in the XY *Sox9* mutants. Together these data suggest that male and female genetic pathways are both main effectors involved in sex determination and that the long-standing dogma of a default female pathway should definitively be revised.

## Introduction

Mammalian sex determination depends on the primary developmental decision of the gonad to differentiate as testis or ovary. The gonad develops as a bipotential organ with the capacity to respond to two different genetic stimuli: the activation of the SRY/SOX9 pathway that induces testicular development, or the expression of the R-spondin1 (RSPO1)/beta-catenin pathway that regulates ovarian differentiation [1]. Indeed in humans and mice, male sex determination is initiated by the expression of the Y-linked gene *SRY* [2], [3], [4]. *Sry* expression in turn activates the transcriptional regulator *SOX9* [5]. Subsequently, SOX9 initiates Sertoli cell differentiation, the supporting cell of the testicular sex cords [6], [7]. Signaling pathways initiated in these cells contribute to the organization of the XY gonads [8], as well as to the differentiation of other testicular cell lineages such as the Leydig steroidogenic cells [9], [10] and the pro-spermatogonia [11], [12], ultimately leading to testis formation and, in turn, male development. In 46,XY patients, loss-of-function mutations in *SRY* and *SOX9* promote male-to-female sex reversal [13], [14], whereas translocations of the *SRY* locus to another chromosome can yield 46,XX patients with female-to-male sex reversal [3]. Loss-of-function mutations [6], [7], [15], [16] and gain-of-function mutations [4], [17], [18] of *Sry* and *Sox9* have been generated in mouse models, showing that *Sry* and *Sox9* are necessary and sufficient to induce testis differentiation and the associated male development. As a consequence, these genes have been considered as the master inducers of testis differentiation and male development.

In the absence of *SRY* (XX individuals), up-regulation of RSPO1, an activator of the WNT/beta-catenin signaling pathway, promotes ovarian differentiation. Mutations in *RSPO1* are responsible for skin disorders and female-to-male sex reversal in 46,XX patients [19]. Similarly, ablation of *Rspo1* in mice yields female-to-male sex reversal and promotes *Sox9* up-regulation correlated with differentiation of Sertoli cells and formation of testis cords at birth [20]. This gonadal dysgenesis yields development of an ovotestis, a gonad displaying both testicular and ovarian regions [20], [21]. *Rspo1* expression in turn activates expression of *Wnt4* [21], another activator of the WNT/beta-catenin signaling pathway involved in ovarian differentiation [22], [23]. When the canonical beta-catenin signaling pathway is activated in XY gonads, this induces male-to-female sex reversal indicating that this pathway acts on top of ovarian differentiation [23]. Indeed, activation of WNT/beta-catenin is required for expression of *Foxl2* [24], a transcription factor involved in folliculogenesis [25], [26] and homeostasis of the ovary [27]. Thus *Rspo1* appears to be the gene instructing the molecular network leading to ovarian development.

Since ablation of *Rspo1* promotes SOX9 expression concomitantly with Sertoli cell differentiation [20], it was assumed that *Sox9* is the sex reversal inducer in XX *Rspo1^KO^* mutants. We now show that i) testicular differentiation occurs in XX *Rspo1^KO^Sox9^cKO^* mutants indicating that neither *Sry* nor *Sox9* are required for female-to-male sex reversals; ii) testicular differentiation also occurs in XY *Rspo1^KO^Sox9^cKO^* mutants indicating that *Rspo1* is required for male-to-female sex reversal in XY *Sox9^cKO^* mutants.

## Results/Discussion

### *Rspo1* is required for ovarian development in XY *Sox9^cKO^* mice

*Sox9* is required for Sertoli cell differentiation, testis formation and male development. Indeed, deletion of *Sox9* in XY *Sox9^fl/fl^; Sf1:cre^Tg/+^*, (referred to as XY *Sox9^cKO^*) triggers male-to-female sex reversal [16]. However the factor(s) inducing sex reversal in XY *Sox9^cKO^* remained to be identified. Given (i) the prominent role of RSPO1, an activator of beta-catenin signaling, in female sex determination [19], and (ii) the fact that ectopic activation of beta-catenin in XY gonads can induce male-to-female sex reversal [23], we hypothesized that *Rspo1* expression induced male-to-female sex-reversal in XY *Sox9^cKO^* gonads. According to this scenario, neither testicular (which is *Sox9*-dependent) nor ovarian (which is *Rspo1*/beta-catenin-dependent) differentiation should occur in XY *Sox9^cKO^* gonads additionally lacking *Rspo1*. To test this hypothesis, we have generated and analyzed double loss-of-function mice (i.e. XY *Rspo1^−/−^; Sox9^fl/fl^; Sf1:cre^Tg/+^*, referred to as XY *Rspo1^KO^Sox9^cKO^*).

**Figure 1:**
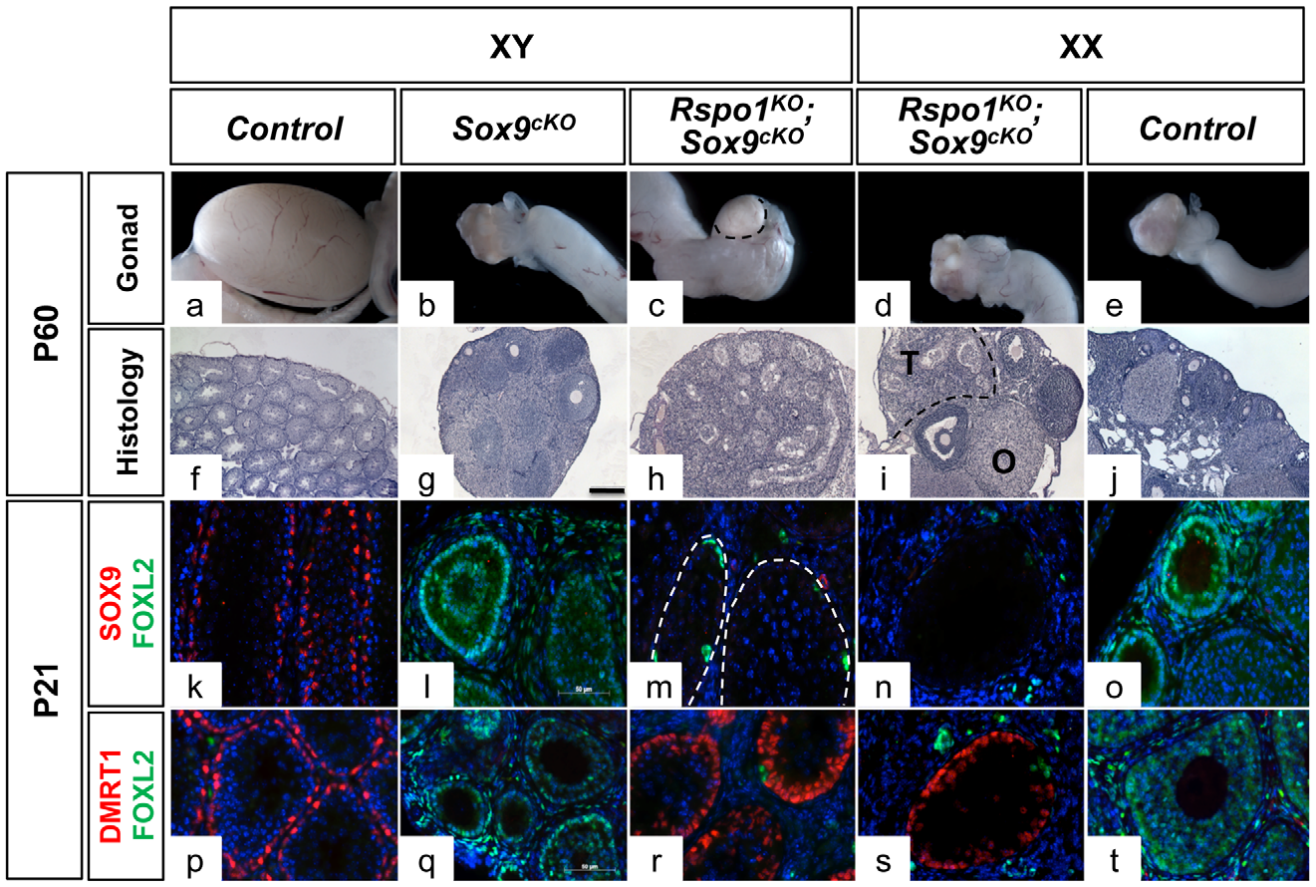
Testicular differentiation in XY and XX *Rspo1^−/−^; Sox9^flox/flox^; Sf1;cre^Tg/+^* (*Rspo1^KO^Sox9^cKO^*) mice. Macroscopic views of gonads of 2 month-old mice show hypoplasic testis and ovotestis development in XY (c) and XX (d) *Rspo1^KO^Sox9^cKO^* mice, respectively. Seminiferous tubules are revealed by PAS histological analysis of XY (h) and XX (i) *Rspo1^KO^Sox9^cKO^* gonadal sections. They are less abundant than in XY controls (f). XY *Sox9^cKO^* gonads (g) develop as ovaries (j). (T: testicular region, O: ovarian region, scale bar: 200 µm). Immunofluorescence of SOX9 (k–o) or DMRT1 (p–t) (a Sertoli cell marker, in red), FOXL2 (k–t) (a follicular cell marker, in green) and DAPI (a nuclear marker in blue) (scale bar, 50 µm). Deletion of *Sox9* with *Sf1:cre* (*Sox9^cKO^*) eliminates SOX9 expression in Sertoli cells (l, m, n), and promotes male-to-female sex reversal in XY *Sox9^cKO^* gonads as highlighted by robust FOXL2 expression (l, q). However, *Sox9* deletion no longer allows ovarian cells differentiation when *Rspo1* is deleted in the XY (m, r) and XX (n, s) *Rspo1^KO^Sox9^cKO^* mice. This is evidenced by the robust expression of DMRT1 in 3 week-old XY (s) and XX (r) mutant gonads and XY controls (p), and the low or absent expression of FOXL2 in these gonads (k, m, n, p, r, s). XY (a, f, k, p) and XX (e, j, o, t) *Rspo1^+/−^; Sox9^flox/flox^* controls, XY *Sox9^cKO^* gonads (b, g, l, q), XY (c, h, m, r) and XX (d, i, n, s) *Sox9^cKO^ Rspo1^KO^* respectively. XX *Rspo1^KO^* and XX *Sox9^cKO^ Rspo1^KO^* gonads appeared similar (see Figure S2B).

Since previous results have shown that sex reversal can appear quite late during fetal development [20], [22], we first analyzed adult stages when the sexual development is likely to be completed. At P60 (postnatal day 60), the anogenital distance in XY *Rspo1^KO^Sox9^cKO^* mice was equivalent to that of XX controls but the internal genitalia contained both male and female organs including oviducts, uterine horns and vaginal tissues, as well as epididymides, vasa deferensia, seminal vesicles and prostate (Figure S1). The XY *Sox9^cKO^* developed as ovaries (Figure 1b, 1g, 1l, 1q and Figure S1), as expected from a previous report [16]. Interestingly, XY *Rspo1^KO^Sox9^cKO^* gonads developed as hypoplastic testes containing well-defined seminiferous tubules as evidenced by histological analysis (Figure 1c, 1h and Figure S1). We next examined whether the supporting cells forming the seminiferous tubules differentiated as granulosa cells, the ovarian supporting cells expressing FOXL2 [25], [28] or as Sertoli cells expressing DMRT1 [29]. In P21 gonads, immunostaining experiments showed that the supporting cells forming the seminiferous tubules in XY *Rspo1^KO^Sox9^cKO^* gonads were DMRT1-expressing Sertoli cells (Figure 1r), even though SOX9 was clearly missing (Figure 1m). However, a few FOXL2-positive granulosa cells were found within the alignment of the Sertoli cells forming the seminiferous tubules (Figure 1m, r) and in a few XY *Rspo1^KO^Sox9^cKO^* mice (3 out of 18), rare and abnormal follicles were observed (Figure S2A). The mixed genetic background of *Rspo1^KO^Sox9^cKO^* mice is a likely factor causing the variation of this phenotype.

Altogether this shows that a genetic pathway activated by RSPO1 is required for the male-to-female sex-reversal of XY *Sox9^cKO^* and indicates that Sertoli cell differentiation and seminiferous tubules formation can occur in the absence of SOX9.

### Sertoli cell differentiation occurs without *Sry* and *Sox9* in XX *Rspo1^KO^* gonads

Our study also allowed us to evaluate the effect of *Sox9* removal in a female-to-male sex reversal context (i.e. in XX *Rspo1^KO^Sox9^cKO^*). Given that homozygous mutations of *Rspo1* promote Sertoli cell differentiation around birth, a process that is associated with *Sox9* up-regulation in these cells [20], we hypothesized that *Sox9* is the inducing factor of testicular differentiation in XX *Rspo1^KO^* mice. If *Sox9* is indeed the main switch for female-to-male sex reversal in XX individuals, one expects an impaired differentiation of Sertoli cell and seminiferous tubules in the absence of both *Rspo1* and *Sox9* in XX *Rspo1^KO^Sox9^cKO^* gonads. Unexpectedly, at P60, these XX double mutants displayed hermaphroditism of the reproductive tracts (Figure S1). Histological analysis revealed that XX *Rspo1^KO^Sox9^cKO^* mice exhibited ovotestes with an extensive presence of sex cords (Figure 1d, 1i and in Figure S2B, f) as do XX *Rspo1^KO^*gonads (shown in Figure S2B, S2e and in previous analyses [20], [21]). Thus, the development of XX *Rspo1^KO^Sox9^cKO^* mouse genitalia is indistinguishable from that of XX *Rspo1^KO^* mice indicating that the additional deletion of *Sox9* in XX *Rspo1^KO^Sox9^cKO^* gonads does not change the fate of XX *Rspo1^KO^* gonads. We next examined whether the supporting cells forming the sex cords differentiated as granulosa cells, the ovarian supporting cells expressing FOXL2 [25], [28], or as Sertoli cells expressing DMRT1 [29]. In three weeks old mice (P21), *Sox9*-depleted cells forming the seminiferous tubules generally lacked the follicular cell marker FOXL2 and instead expressed DMRT1 (Figure 1n, 1s). These data clearly indicate that Sertoli cell, seminiferous tubule and testis differentiation can occur in the absence of *Sry* and *Sox9* in XX *Rspo1^KO^* gonads.

### Steroidogenic cells are present in *Rspo1^KO^Sox9^cKO^* embryonic gonads

Previous studies clearly show that the development of male genitalia depends on androgens secreted by the embryonic testis [30]. In XX *Rspo1^KO^* gonads, steroidogenic cells appear before Sertoli cell differentiation [20], [31] and this was also observed in *Wnt4^KO^* gonads [22], [32], *Wnt4* being up-regulated upon *Rspo1* expression in XX gonads [20], [21]. In addition, lack of *Wnt4* expression was shown to allow ectopic migration of steroidogenic cells from the neighboring adrenals into gonads [32], [33] and subsequent androgen synthesis [34], which explains the development of male genitalia in these mutants. When investigating whether steroidogenic cells were present in XX and XY *Rspo1^KO^Sox9^cKO^* gonads, we found that *P450Scc*, a gene encoding for a precursor involved in androgen synthesis was expressed at 14.5 d*pc* in XY controls, XY and XX *Rspo1^KO^Sox9^cKO^* gonads and XX *Rspo1^KO^* gonads, but not in XX controls (Figure S3A). However, *Cyp21*, a marker for adrenal cells [35], was not strongly expressed in *Rspo1^KO^Sox9^cKO^* gonads at 13.5 d*pc* (Figure S3A), suggesting that the steroidogenic cells in *Rspo1^KO^Sox9^cKO^* gonads, did not come from the arenals or, alternatively, have undergone reprogramming as Leydig cells. Whatever the situation, it is likely that male hormones synthesized in the developing mutant gonads can contribute to stimulate epididymides, vasa deferentia and seminal vesicles development.

### Delayed testicular formation in *Rspo1^KO^Sox9^cKO^* mice

**Figure 2:**
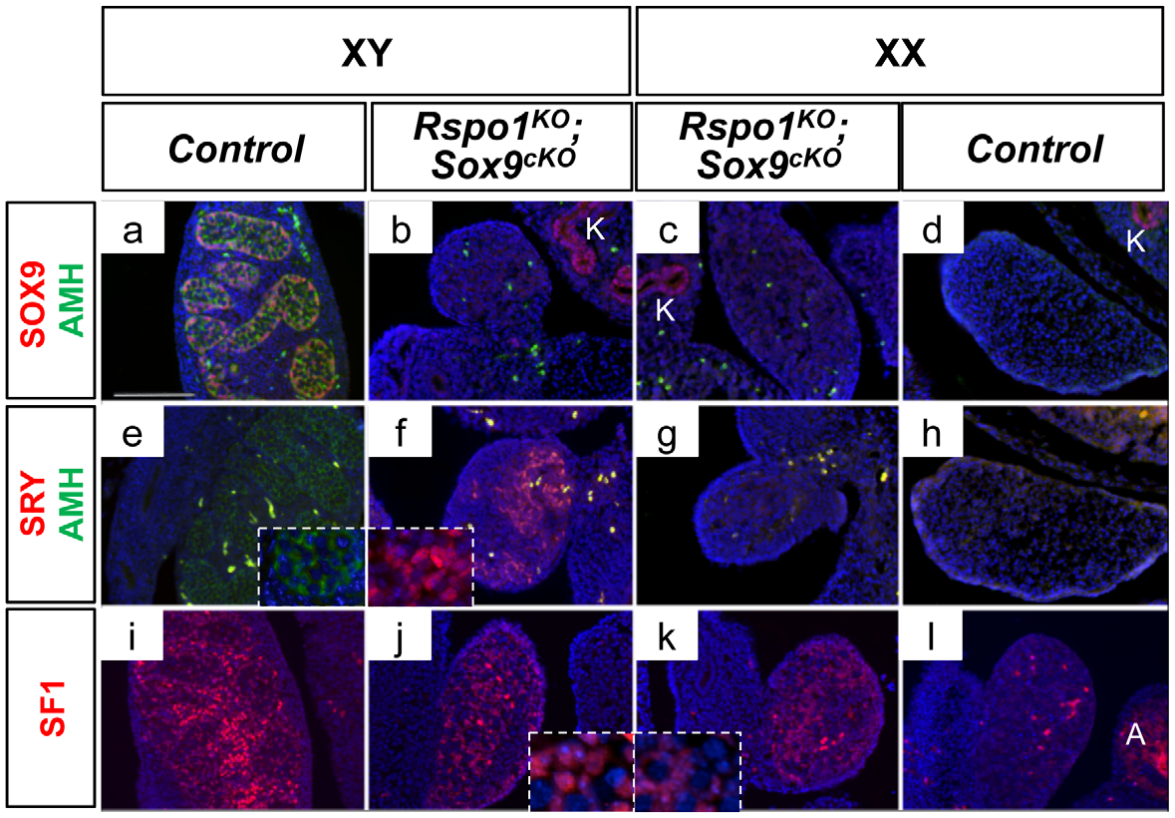
Non-differentiated XY and XX *Rspo1^KO^Sox9^cKO^* gonads at 13.5 d*pc*. Immunofluorescence of SOX9 (Sertoli cell marker, in red) and AMH (Sertoli cell marker green) (a–d), AMH (Sertoli cell marker, in green) and SRY (pre-Sertoli and Sertoli cell marker in red) (e–h) and SF1 (undifferentiated supporting cell, Sertoli and Leydig cell marker) (i–l). Counterstain is DAPI (in blue). Lack of SOX9 and AMH expression in XY (b) and XX (c) *Rspo1^KO^Sox9^cKO^* gonads shows that Sertoli cell differentiation did not occur at 13.5 d*pc*. Note that the kidneys (K) are positive for SOX9. This is accompagnied with the maintenance of SRY expression in the XY *Rspo1^KO^Sox9^cKO^* gonads (f) whereas SRY expression has ceased in XY controls (e). SF1 expression is maintained in absence of Sertoli cells differentiation in XY and XX *Rspo1^KO^Sox9^cKO^* gonads (j and k respectively) (scale bar: 100 µm). Note that SF1 is also expressed in steroidogenic cells of the adrenals (A). XY (a, e, i) and XX (d, h, l) *Rspo1^+/−^; Sox9^flox/flox^* controls, XY (b, f, j) and XX (c, g, k) *Sox9^cKO^ Rspo1^KO^* respectively.

We next investigated the timing of testicular cord formation in XY and XX *Rspo1^KO^Sox9^cKO^* gonads. In wild-type embryos, the earliest morphological sign of testis development occurs at 12.0–12.5 d*pc* when testis cord are formed [36]. Accordingly at 13.5 d*pc*, the testis cords were highlighted in XY controls by the prominent expression of SOX9 and AMH, two markers of Sertoli cells (Figure 2a). In contrast, *Rspo1^KO^Sox9^cKO^* gonads did not show a clear testicular organization as they lack AMH at 13.5 d*pc* (Figure 2b, 2c, 2f, 2g). As AMH synthesis and secretion by Sertoli cells promotes the elimination of the female reproductive tract during embryogenesis [37], the absence of AMH in *Rspo1^KO^Sox9^cKO^* gonads provides an explanation for the maintenance of the Mullerian derivatives (oviducts, uterine horns and vaginal tissues) in these mutant mice. In addition, Sertoli cell differentiation is delayed in gonads lacking both *Sox9* and *Rspo1*, as indicated by the maintenance of SRY expression, in the XY *Rspo1^KO^Sox9^cKO^* gonads at 13.5 d*pc* (Figure 2f), a stage at which SRY expression has already ceased for one day in the control situation [38], [39], [40]. Along these lines, the maintenance of SF1 expression in XX *Rspo1^KO^Sox9^cKO^* gonads at 13.5 d*pc* (Figure 2j, 2k), a factor whose expression is normally down-regulated between 13.5 and 16.5 d*pc* in the ovary [41] (Figure 2l), also suggests that the XX *Rspo1^KO^Sox9^cKO^* gonads are still undifferentiated or have differentiated as testis. However, with respect to the latter, the absence of AMH expression shows that no Sertoli cell differentiation has occurred (Figure 2c, 2g). Altogether these data indicate that the *Rspo1^KO^Sox9^cKO^* gonads are still undifferentiated at 13.5 d*pc*.

The first signs of Sertoli cell differentiation appeared at 16.5 d*pc* in *Rspo1^KO^Sox9^cKO^* gonads, with some rare DMRT1-positive cells in comparison to XY controls (Figure S3B). Then, few rudimentary testis cords were observed around 17.5 d*pc* (Figure S3B). At P0, Sertoli cells aligned to form sex cords as evidenced by the localization of DMRT1 positive-cells (Figure S4A c, d). Quantitative PCR experiments further confirmed that *Dmrt1* expression was strongly expressed in XY *Rspo1^KO^Sox9^cKO^* gonads and weakly in XX *Rspo1^KO^Sox9^cKO^* gonads at P0, highlighting that more Sertoli cells were present in XY *Rspo1^KO^Sox9^cKO^* gonads in comparison to XX *Rspo1^KO^Sox9^cKO^* gonads (Figure S4C). In addition, some FOXL2-positive cells were also detected in *Rspo1^KO^Sox9^cKO^* gonads (Figure S4A c, d). However, quantitative PCR experiments showed that *Foxl2* expression was significantly reduced in comparison to XX control or XY *Sox9^cKO^* gonads (Figure S4B) as expected for a gonad developing as ovotestis or testis.

**Figure 3:**
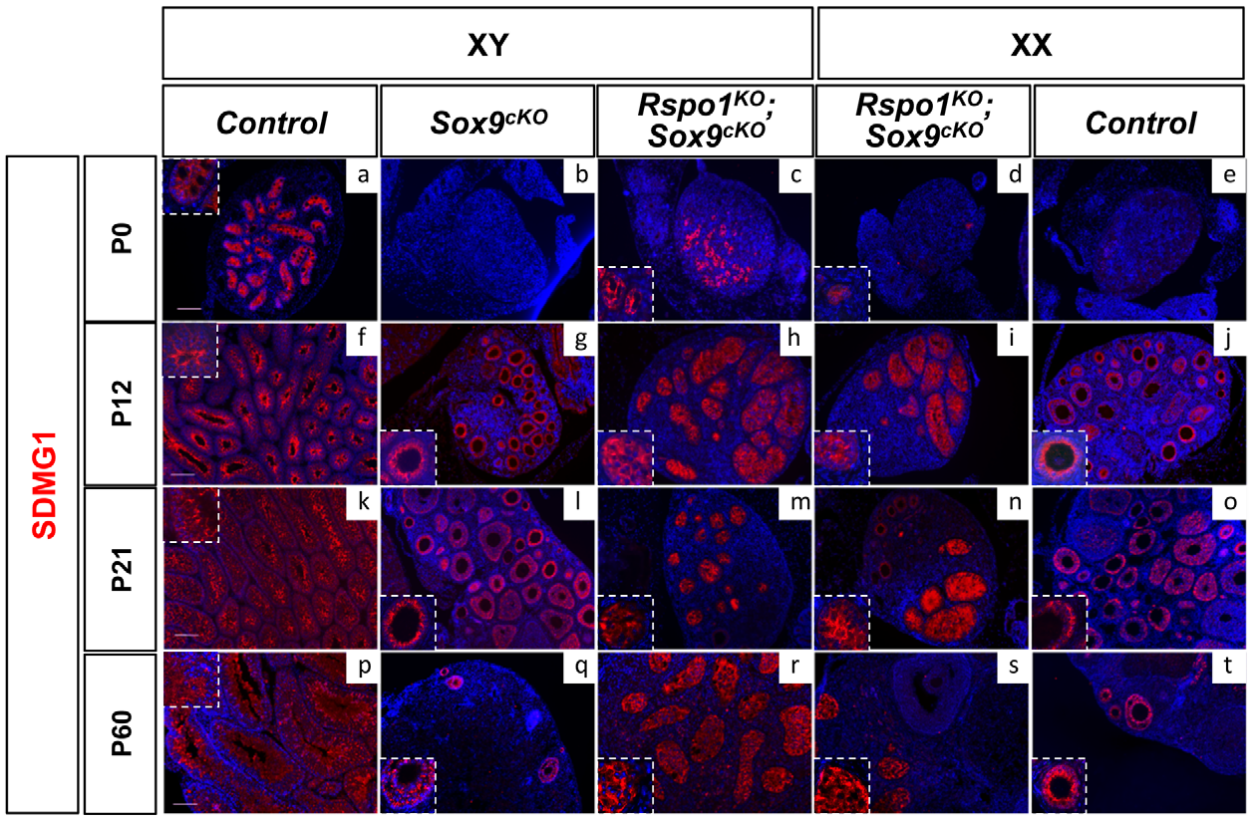
Post-natal development of sex cords in XY and XX *Rspo1^KO^Sox9^cKO^* mice. Immunofluorescence of SDMG1 (in red). Counterstain is DAPI (in blue). SDMG1 is expressed in Sertoli cells (XY controls a, f, k, p) and in follicular cells of growing ovaries as evidenced at P12 onwards (j, o, t). Sertoli cells are present and formed sex cords in both XY and XX *Rspo1^KO^Sox9^cKO^* gonads, with more developing sex cords in XY *Rspo1^KO^Sox9^cKO^* testis (c, h, m, r) in comparison to XX *Rspo1^KO^Sox9^cKO^* ovotestis (d, i, n, s). At P12, the sex cords are fully developed in both XY (h) and XX (i) *Rspo1^KO^Sox9^cKO^* mice. In XY *Sox9^cKO^* (b, g, l, q) and XX control (e, j, o, t) gonads, ovarian follicles express SDMG1 at P12, P21 and P60. At these stages, SDMG1 is also expressed in the follicles of the XX double mutant ovotestes (see n) and in XY double mutant follicles when they develop (scale bars: 100 µm). XY (a, f, k, p) and XX (e, j, o, t) *Rspo1^+/−^; Sox9^flox/flox^* controls, XY *Sox9^cKO^* gonads (b, g, l, q), XY (c, h, m, r) and XX (d, i, n, s) *Rspo1^KO^ Sox9^cKO^* gonads respectively.

**Figure 4:**
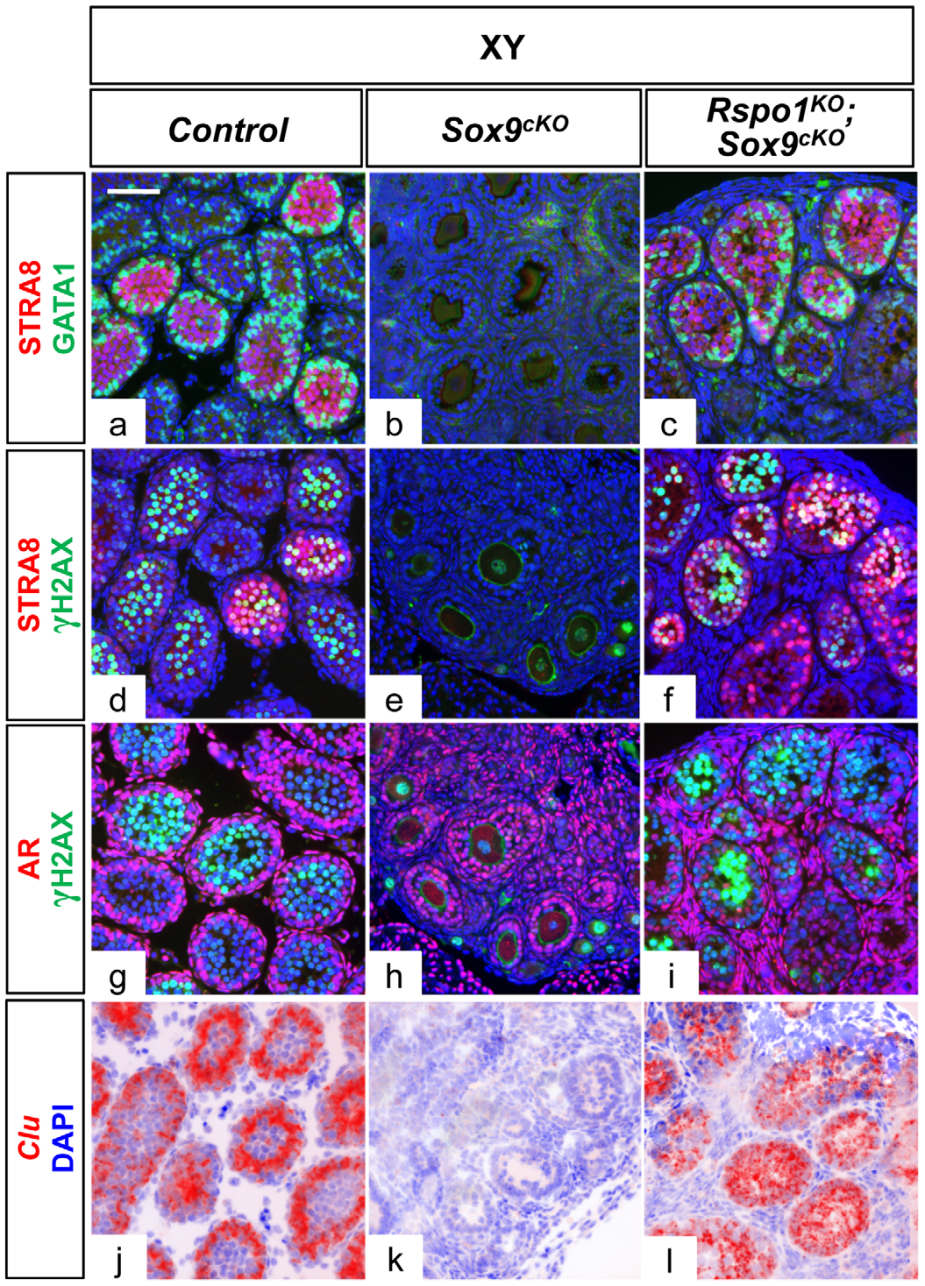
Sertoli cells support germ cell differentiation in XY *Rspo1^KO^Sox9^cKO^* gonads. Immunofluorescence (a–i) of GATA1 (Sertoli cell marker, in green), AR (Androgen Receptor) (Sertoli, peritubular myoid and Leydig cell marker, in red), STRA8 (a premeiotic marker, in red), and γH2AX (a meiotic marker, in green) at P10. Counterstain is DAPI (in blue). In situ hybridization (j–l) using a probe for *Clu* transcripts, another marker for mature Sertoli cells, illustrated as computer–generated bright field superimpositions of the blue counterstain (DAPI) with the hybridization signal (red false color). GATA1, AR and *Clu* expression show that the Sertoli cells mature in XY controls (a, g, j) and XY *Rspo1^KO^Sox9^cKO^* testes (c, i, l), and are able to support germ cell differentiation until meiosis initiation as revealed by STRA8 (a, c, d, f) and γH2AX (d, f, g, i) expression. Note that both Sertoli, peritubular myoid and Leydig cells of XY *Sox9^cKO^* mutant gonads normally expressed AR (h). (scale bars: 50 µm). XY (a, d, g, j) *Rspo1^+/−^; Sox9^flox/flox^* controls, XY *Sox9^cKO^* gonads (b, e, h, k) and XY *Rspo1^KO^Sox9^cKO^* (c, f, i, l) gonads.

We then studied SDMG1 expression, a cytoplasmic marker of Sertoli cells and of granulosa cells when follicles form (Best et al. 2008). Using this marker, sex cords were evident at P0 (Figure 3c, 3d and Figure S5c, S5d) and, at puberty (P12), development of the seminiferous tubules appeared complete in *Rspo1^KO^Sox9^cKO^* gonads (Figure 3h, 3i and Figure S5h, S5i). At puberty, androgen receptor (AR) immunostaining indicated that, in addition to Sertoli cells, peritubular myoid and Leydig cells were also present both in XY *Rspo1^KO^Sox9^cKO^* testes (Figure 4i) and in testicular parts of the XX *Rspo1^KO^Sox9^cKO^* ovotestes (data not shown). In addition, follicle development appeared at P12 in XX *Rspo1^KO^Sox9^cKO^* ovotestes and XX control ovaries (Figure S2B d, f). Together our results indicate that seminiferous tubule development is delayed in the absence of *Sox9* and *Rspo1*, thereby explaining the small size of the XY *Rspo1^KO^Sox9^cKO^* testes (Figure 1c).

### SOX9-negative Sertoli cells can support germ cell differentiation until initiation of meiosis

We next investigated whether the Sertoli cells that differentiate in the *Rspo1^KO^Sox9^cKO^* gonads can support germ cell differentiation. Since XX germ cells cannot survive in a testicular environment [42], [43], the analysis was only carried out in XY *Rspo1^KO^Sox9^cKO^* gonads. In the normal fetal testis, following Sertoli cell differentiation, prospermatogonia become quiescent from 14.5 d*pc* and express the multipotency marker *Oct4* until 17.5 d*pc* [44]. At that time, *Cyp26b1*, a protein involved in retinoic acid degradation, contributes to prevent germ cells from entering meiosis [45], [46]. As expected, the majority of prospermatogonia in XY *Rspo1^KO^Sox9^cKO^* gonads expressed *Oct4* at 14.5 d*pc* (Figure S6o). Nonetheless, some cells had already committed to meiosis (Figure S6k) and expressed the meiotic marker *Stra8* [47], possibly because of the low level of *Cyp26b1* expression in XY *Rspo1^KO^Sox9^cKO^* gonads (Figure S6g). The reduced level of *Cyp26b1* expression is however not sufficient to allow all germ cells to enter meiosis in XY *Rspo1^KO^Sox9^cKO^* gonads.

At P10, GATA1, Androgen Receptors (AR) and Clusterin (*Clu*) were normally expressed in Sertoli cells of XY *Rspo1^KO^Sox9^cKO^* gonads (Figure 4c, 4i, 4l), suggesting that these cells have acquired their identity and may be capable to support spermatogenesis. Accordingly, XY germ cells had committed to meiosis at P10, as assessed by immunodetection of the pre-meiotic and meiotic markers STRA8 and γH2AX, respectively (Figure 4c, 4f, 4i). However, later stages of spermatogenesis cannot however be analyzed, as hypoplasia of germ cells occurred within the seminiferous tubules of adult XY *Rspo1^KO^Sox9^cKO^* gonads (Figure 1h and Figure S1m), most likely because of cryptorchidism.

### *Sox8* and *Sox10* are expressed in the absence of *Sox9*

**Figure 5:**
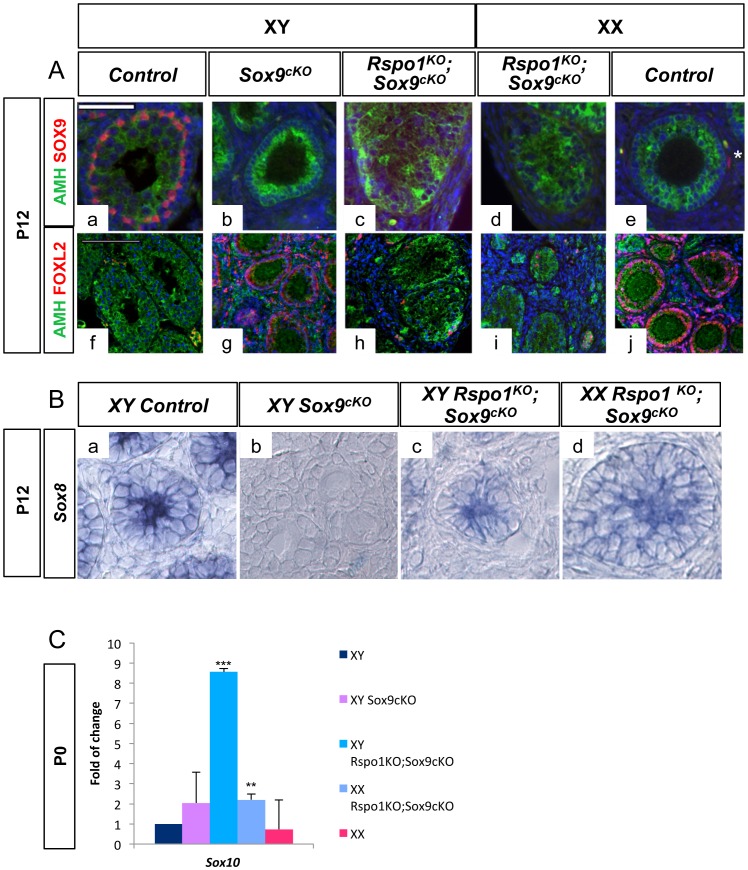
AMH and SOX genes are expressed in XY and XX *Rspo1^KO^ Sox9^cKO^* gonads. A- AMH expression in absence of SOX9. Immunofluorescence of SOX9 (in red) and AMH (in green). Counterstain is DAPI (in blue). SOX9 and AMH are synthetised in Sertoli cells of the testis (a, f). SOX9 is expressed in theca cells (white star in e) and AMH in follicular cells of the ovary at P12 (e, j). Deletion of *Sox9* with *Sf1:cre* eliminates SOX9 expression in *Sf1:cre* positive cells of the gonads, which are Sertoli cells in XY (c) and XX (d) *Rspo1^KO^Sox9^cKO^* gonads and theca cells of the ovarian region of XX *Rspo1^KO^Sox9^cKO^* gonads (d) and XY *Sox9^cKO^* gonads (b). AMH expression is observed in Sertoli cells of the XY (c, h) and XX (d, i) *Rspo1^KO^Sox9^cKO^* gonads even the absence of SOX9. (scale bar: 50 µm). Immunofluorescence of FOXL2 (in red) and AMH (in green). Counterstain is DAPI (in blue). Most of the AMH positive cells in the testicular cords of *Rspo1^KO^Sox9^cKO^* gonads (h, i) are negative for FOXL2 indicating that they are not granulosa cells, some AMH/FOXL2 positive cells were observed outside of these cords indicating that they are granulosa cells (h, i). (scale bar: 100 µm). XY (a, f) and XX (e, j) *Rspo1^+/−^;Sox9^flox/flox^* controls, XY *Sox9^cKO^* gonads (b, g), XY (c, h) and XX (d, i) *Rspo1^KO^ Sox9^cKO^* gonads respectively. B- *Sox8* is expressed in XY and XX *Rspo1^KO^ Sox9^cKO^* gonads. *In situ* hybridization of *Sox8* transcripts. *Sox8* is expressed in Sertoli cells at P5 in XY control (a), XY (c) and XX (d) *Rspo1^KO^Sox9^cKO^* gonads, but not in XY *Sox9^cKO^* ovaries (b). (a) XY *Rspo1^+/−^; Sox9^flox/flox^* controls, (b) XY *Sox9^cKO^* gonads, XY (c) and XX (d) *Rspo1^KO^ Sox9^cKO^* gonads respectively. C- *Sox10* is expressed in XY and XX *Rspo1^KO^ Sox9^cKO^* gonads. QPCR analysis shows that *Sox10* is significantly up-regulated both in XY and XX *Rspo1^KO^Sox9^cKO^* gonads, when compared to XY controls. The differences between XY controls and XY *Sox9^cKO^* are not significant.

Interestingly, we found that AMH was expressed in Sertoli cells of both XX and XY *Rspo1^KO^Sox9^cKO^* gonads at P12 (Figure 5A). Given that (i) *Amh* is a target-gene of SOX9 [48], [49], (ii) *Amh* expression can be induced by SOX8 [50] and SOX10 [51], and (iii) *Sox10* ectopic up-regulation in XX gonads can induce testis differentiation [51], we hypothesized that a *Sox* factor distinct from *Sox9* could have induced late AMH expression in *Rspo1^KO^Sox9^cKO^* gonads and delayed testicular differentiation. In agreement with this possibility, expression of both *Sox8* and *Sox10* was activated in *Rspo1^KO^Sox9^cKO^* mutants at P12 and P0 respectively (Figure 5B, 5C). Previous data have shown that *Sox8* becomes crucial from 14.5 d*pc* onwards, for the maintenance of testis development [52], suggesting that Sertoli cell differentiation can be induced by *Sox* genes other than *Sox9* during late embryogenesis. However, the function of these *Sox* genes during late development is likely not sufficient to replace the role of Sox9 in early Sertoli cells development, thus leading to the formation of an hypoplastic testis, as is the case in the XY *Rspo1^KO^Sox9^cKO^* mice. To date, the only factors that have been shown to be able to induce Sertoli cell differentiation are *Sox* genes [51], [53], while other factors such as *Dmrt1* are required after birth (P7) for the maintenance of Sertoli cell identity [54]. Further studies are required to address whether DMRT1 is able to allow Sertoli cell differentiation from undifferentiated supporting cells. Given that *Sox9* expression is controlled by *Wt1* when *Sry* expression has ceased [55], we can speculate that *Wt1* might also be involved in *Sox8* and *Sox10* expression in these mutants. Furthermore, FGF9 or PGD2, two secreted factors synthesized in the undifferentiated gonads, [56], [57] can also contribute to Sertoli cell differentiation [58], [59], [60]. Whether *Wt1*, PGD2 or FGF9 signaling also regulate *Sox8* and *Sox10* remains to be investigated.

In addition, when XX and XY *Rspo1^KO^Sox9^cKO^* gonads are compared at the same stage, XY gonads always appear more masculinized than XX gonads (Figure 1, Figure 3, Figure S1, Figure S3, Figure S5), because they contain more sex cords/seminiferous tubules. At a molecular level, the main difference between XX and XY *Rspo1^KO^Sox9^cKO^* gonads is the expression of SRY in XY gonads (Figure 2). Indeed, SRY expression is maintained in XY *Rspo1^KO^Sox9^cKO^* gonads at 13.5 d*pc*, while at this time point its expression has ceased in XY control gonads. This suggests that SRY participates in the masculinization of the XY *Rspo1^KO^Sox9^cKO^* gonads by inducing the expression of genes other than *Sox9* to promote sex cord formation.

**Figure 6:**
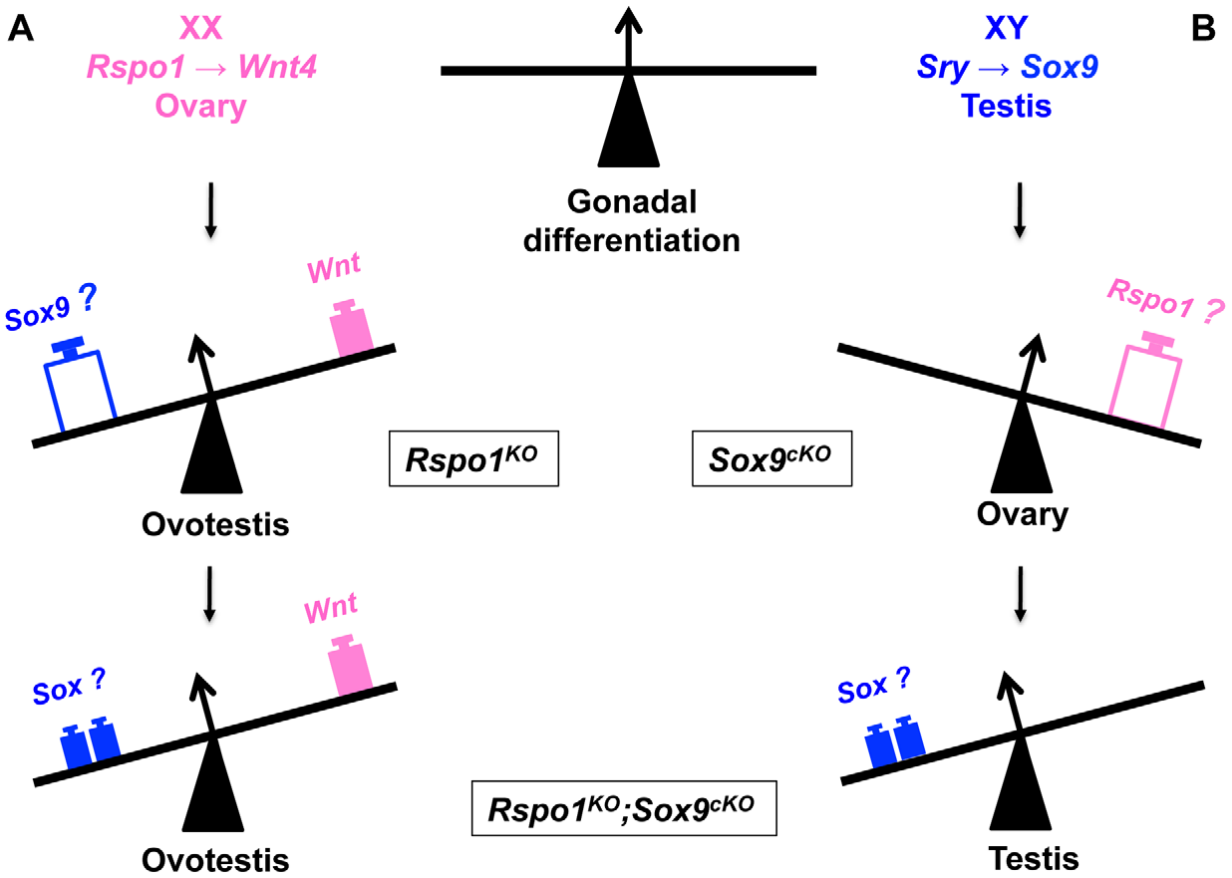
Opposing function of SOX and RSPO1 signaling in the fate of the gonad. A- In XX gonads, RSPO1 activates WNT/beta-catenin signaling to promote ovarian differentiation. Ablation of *Rspo1* results in partial sex reversal with ovotestis development, which coincides with *Sox9* expression. However additional deletion of *Sox9* in the XX *Rspo1* ^KO^ (i.e., *Rspo1^KO^Sox9^cKO^*) still allows ovotestis formation, implying that *Sry* and *Sox9* are not required for testicular differentiation in female-to-male sex reversal. B- In XY gonads, whereas *Sox9* deletion triggers ovarian development, additional deletion of *Rspo1* in XY *Rspo1^KO^Sox9^cKO^* gonads restores testis development. This is associated with the expression of other SOX genes like SOX 8 and SOX10, other masculinising factors.

In summary, here we have shown that (i) both SRY and SOX9 are dispensable for female-to-male sex reversal in *Rspo1^KO^*, (ii) RSPO1 signaling is required for male-to-female sex reversal in *Sox9^cKO^*, (iii) Sertoli cell differentiation and seminiferous tubule formation can occur in the absence of SOX9, possibly because of a functional redundancy with other SOX proteins such as SOX8 and SOX10. Indeed, ectopic expression of *Sox10* in XX gonads has been shown to promote testicular differentiation [51]. Altogether these data show that SRY and SOX9 are not the only masculinizing factors, since other SOX proteins can induce female-to-male sex reversal in pathophysiological conditions (Figure 6). Following Sertoli cell differentiation, DMRT1 expression becomes required for the maintenance of the Sertoli cells and the testicular tissue [54]. Furthermore, our data suggest that the feminizing factors remaining in *Rspo1^KO^* mice can be overtaken by SOX proteins, when they are activated in XX gonads. Testicular differentiation in the absence of *Rspo1* expression in XY *Sox9^cKO^* gonads was unexpected since female development is thought to be a default pathway [61], [62]. Our results imply that instead the female pathway needs to be activated. Therefore our genetic study suggests that mammalian sex determination is regulated by a finely tuned balance between two main factors [56], [63], which are the SOX genes on the one hand and the RSPO1/WNT/beta-catenin signaling pathway on the other hand.

## Materials and Methods

### Mouse strains and genotyping

The experiments here described were carried out in compliance with the relevant institutional and French animal welfare laws, guidelines and policies. They have been approved by the French ethics committee (Comité Institutionnel d'Ethique Pour l'Animal de Laboratoire; number NCE/2011-12). All mouse lines were kept on a mixed 129SV/C57BL6/J background. *Rspo1^−/−^*, *Sox9^flox/flox^* mice and *Sf1:cre^Tg/+^* transgenic mice (a kind gift from Keith Parker) were described previously [64]. *Rspo1^−/−^* male were mated with *Sox9^flox/flox^*; *Sf1:cre^Tg/+^* female [16] to obtain *Rspo1^+/−^; Sox9^flox/+^; Sf1:cre^Tg/+^* females and *Rspo1^+/−^; Sox9^flox/+^* males. Matings between these littermates allowed us to generate *Rspo1^−/−^; Sox9^fl/fl^; Sf1:cre^Tg/+^* mice, referred to as *Rspo1^KO^ Sox9^cKO^* mice, and controls. Gonad samples were collected from timed pups (day of birth = P0). Genotyping was performed as described [7], [20], [64] using DNA extracted from tail tip or ear biopsies of mice. The presence of the Y chromosome was determined as described previously [65]. *Pa*x6 primer set (5′-GCAACAGGAAGGAGGGGGAGA-3′; 5′-CTTTCTCCAGAGCCTCAATCTG-3′) was included in each PCR reaction as an internal control.

### Histological analysis

Urogenital organs were dissected, fixed in Bouin's solution overnight, and then embedded in paraffin. Microtome sections of 5 µm thickness were stained with periodic acid Schiff (PAS) or hematoxylin and eosin (H&E) according to standard procedures. Pictures were taken with an Axiocam mrm camera (Zeiss) and processed with Adobe Photoshop.

### Immunological analyses

Gonad samples were fixed with 4% (w/v) paraformaldehyde overnight and then processed for paraffin embedding. Gonad samples for cryosections were successively fixed for 2 hours in 4% (w/v) paraformaldehyde, washed in cold phosphate-buffered saline (PBS), equilibrated in 10% (w/v) sucrose solution during 3 hours, then in 30% (w/v) sucrose solution overnight at 4°C, embedded in Cryomount (Histolab) and stored at −80°C. For paraffin-embedded and Cryomount-embedded samples, sections of 5 and 8 µm thickness were processed, respectively. The following dilutions of primary antibodies were used: AMH/MIS (C-20, sc-6886, Santa Cruz), 1∶200; AR (sc-816, SantaCruz), 1∶100; DMRT1 (kindly provided by David Zarkower), 1∶500; FOXL2 (ab5096, Abcam), 1∶250; γH2AX (U5-636, Upstate), 1∶500; GATA1 (sc-265, SantaCruz), 1∶50; SDMG1 (a kind gift from Ian Adams) 1∶2000; SF1 (kindly provided by Ken Morohashi) 1∶1500; SOX9 (HPA-001758, Sigma) 1∶250 and SRY [59], [66] 1∶100, STRA8 (ab49602, Abcam), 1∶100. Counterstain with 4′,6-diamidino-2-phenylindole (DAPI) was used to detect nuclei (in blue). Fluorescent studies were performed with a motorized Axio ImagerZ1 microscope (Zeiss) and pictures were taken with an Axiocam mrm camera (Zeiss) and processed with Axiovision LE.

### *In situ* hybridization

Embryos were fixed with 4% paraformaldehyde (PFA) in 1×PBS at 4°C overnight. Further processing of embryos and *in situ* hybridization were carried out essentially as described [67]. *Sox9* riboprobe was synthesized according to [68] and *Sox8* to [69], *P450scc*, *Stra8 and Oct4* riboprobes synthesis were carried out as described previously [20]. *In situ* hybridisation (ISH) with digoxigenin–labelled probes was performed as described [70], using 10 µm–thick cryosections. Each experiment was repeated on at least two gonads. Post–hybridization washes were done in 100 mM maleic acid pH7.5, 150 mM NaCl, 0.1% (v/v) tween–20 (MABT). To increase the sensitivity, 5% (v/v) polyvinyl alcohol (Sigma) was added to the staining solution [71]. Nuclei were counterstained with DAPI diluted in the mounting medium at 10 µg/ml (Vectashield, Vector laboratories). ISH signals corresponding to *Clu*-positive cells were converted into a red false color on the merged pictures. The plasmids containing *Lgals1* (366 bp–long; exons 2–4; MGI:96777) or *Clu* (942 bp–long; exons 5–9; MGI:88423) cDNA fragments were linearized and used as a templates for the synthesis of the sense or antisense riboprobes.

### Quantitative PCR analysis

Individual gonads were dissected in PBS from P0 animals (day of birth) and immediately frozen at −80°C. RNA was extracted using the RNeasy Qiagen kit, and reverse transcribed using the RNA RT–PCR kit (Stratagene). Primers and probes were designed at Roche Assay design center (https://www.rocheappliedscience.com/sis/rtpcr/upl/adc.jsp). Primers are 5′-TCCTCCTCAGACCGCTTTT-3′ and 5′-CCTGGTTCATCATCGCTAATC-3′ (probe 95) for *Hprt1*, and 5′-ATGTCAGATGGGAACCCAGA-3′ and 5′-GTCTTTGGGGTGGTTGGAG-3′ (probe 21) for *Sox10*, 5′-aagaagtgcagcctgattgc-3′ and 5′-ggtggctgatacccagttct-3′ (probe 40) for *Dmrt1*, and 5′-ggcgtcgtgaactcctaca-3′ and 5′-tgcagatgatgtgcgtgag-3′ (probe 51) for *Foxl2*. All real-time, quantitative, PCR assays (QPCR) were carried out using the LC-Faststart DNA Master kit Roche, according to the manufacturer's instructions. QPCR was performed on cDNA from one gonad and compared to a standard curve. QPCR were repeated at least twice. Relative expression levels of each sample were quantified in the same run, and normalized by measuring the amount of *Hprt1* cDNA (which represents the total amount of gonadal cells).

### Statistical analysis

For each genotype (n = 6 individuals), the fold-change was the mean normalized expression levels divided by the mean normalized expression levels of the XY samples considered as the reference. Graphs illustrate fold-changes +1 s.e.m. The results were analyzed using Graphpad for statistical significance that was assessed using one-way ANOVA followed by Tukey-Kramer post-test for selected pairs of genotypes. Asterisks indicate : * p<0.05, ** p<0.01 and *** p<0.001.

## Supporting Information

Figure S1 Testicular differentiation in XY and XX *Rspo1^KO^Sox9^cKO^* mice. External genitalia of XX control mice (e) is similar to XY *Sox9^cKO^* (b), XY and XX *Rspo1^KO^Sox9^cKO^* mice (c and d respectively) at 2 months of age. The internal genitalia of XY and XX *Rspo1^KO^Sox9^cKO^* mice (h, i) show epididymides (E), vasa deferentia (VD) and seminal vesicles (SV), as in XY males (f) but also uterine horns (U) and oviducts (Ovi) as in XX controls (j) or XY *Sox9^cKO^*mice (g). PAS stained histological sections of XY and XX *Rspo1^KO^Sox9^cKO^* gonads (m, n) show seminiferous tubules lacking germ cells because hypoplasia of germ cells occurred in these tubules. XY *Sox9^cKO^* gonads (l) are similar to ovaries (o) (scale bar, 50 µm). XY (a, f, k) and XX (e, j, o) *Rspo1^+/−^;Sox9^flox/flox^*controls, XY *Sox9^cKO^* gonads (b, g, l), XY (c, h, m) and XX (d, i, n) *Rspo1^KO^Sox9^cKO^* gonads respectively.
(TIF)

Figure S2 A- XY *Rspo1^KO^ Sox9^cKO^* gonad containing a single follicle. XY *Rspo1^KO^ Sox9^cKO^* gonad with a single grossly large follicle was located near the entrance of the oviduct (a). This follicle contained three oocytes and was observed in XY *Rspo1^KO^ Sox9^cKO^* gonads on rare occasions (b). B- Comparison of XX *Rspo1^KO^*and XX *Rspo1^KO^ Sox9^cKO^* gonads. Immunofluorescence detection of SDMG1 in Sertoli cells (cytoplasmic) of XX *Rspo1^KO^* (b) and XX *Rspo1^KO^ Sox9^cKO^* (c) gonads at P0. Some sex cords are clearly visible in XX *Rspo1^KO^* and XX *Rspo1^KO^Sox9^cKO^* in contrast to XX control gonads (a). Histological analysis of XX *Rspo1^KO^* and XX *Rspo1^KO^ Sox9^cKO^* gonads at P12 and P21 (e, h and f, i respectively) show the presence of seminiferous tubules and follicles in comparison to XX controls containing only follicles (d, g). Empty and filled arrowheads indicate testis cords and follicles, respectively.
(TIF)

Figure S3 A-Expression of the steroidogenic marker *P450Scc* and *Cyp21* in XY *Rspo1^KO^Sox9^cKO^* gonads. Whole-mount *in situ* hydridization of gonads at 14.5 d*pc* and 13.5 d*pc*. *P450Scc* is expressed in XY *Rspo1^KO^Sox9^cKO^* gonads (b), and in XY controls (a) but not in XX controls (e). *P450Scc*, was expressed in cells at the anterior part of the XX *Rspo1^KO^Sox9^cKO^* and XX *Rspo1^KO^* gonads (c and d respectively) at 14.5 d*pc. Cyp21* was strongly expressed in the adrenals (f, g, h, i, j), whereas no signal was detected in the gonads at 13.5 d*pc* (f, g, h, i, j). Ad: adrenal, G: gonad, K: kidney. B-Delayed testicular cords formation in XY and XX *Rspo1^KO^Sox9^cKO^* gonads. Haematoxylin and eosin stained histological sections of XY and XX *Rspo1^KO^Sox9^cKO^* gonads at 17.5 d*pc* show that some sex cords are forming in the XY *Rspo1^KO^Sox9^cKO^* (b) gonads in contrast to the XY controls (a) containing already formed sex cords. In the littermates XX *Rspo1^KO^Sox9^cKO^* and *Rspo1^KO^* gonads (c and d respectively), no sex cords were observed at this stage (scale bar, 10 µm). Insets in a and b show AMH (Red) and DMRT1 (green), two markers of Sertoli cells highlighting sex cords at 16.5 d*pc*. Sex cords were rare in XY *Rspo1^KO^Sox9^cKO^* gonads and absent from XX *Rspo1^KO^Sox9^cKO^* gonads at this stage.
(TIF)

Figure S4 Detection of DMRT1 and FOXL2 in XY and XX *Rspo1^KO^Sox9^cKO^* gonads at P0. A- Immunofluorescence analysis of DMRT1 and FOXL2 in XY and XX *Rspo1^KO^Sox9^cKO^* gonads at P0. DMRT1 (red), a marker of postnatal Sertoli cells, was detected in the XY control (a), XY *Rspo1^KO^Sox9^cKO^* and XX *Rspo1^KO^Sox9^cKO^* gonads (c and d respectively) while in XY *Sox9^cKO^* (b) and XX (e) control gonads DMRT1 was not detected. The ovarian protein FOXL2 (green) was detected in XY *Sox9^cKO^* and XX control gonads, and few FOXL2-positive cells (granulosa cells) were found within the XY *Rspo1^KO^Sox9^cKO^* (c) and XX *Rspo1^KO^Sox9^cKO^* (d) gonads. B- qPCR anaylsis of *Foxl2* in XY and XX *Rspo1^KO^Sox9^cKO^* gonads at P0. *Foxl2* is significantly up-regulated in the XY *Sox9^cKO^* compared to XY and XX *Rspo1^KO^Sox9^cKO^* indicating that ablation of *Rspo1* leads to reduced *Foxl2* expression at a transcriptional level. C- qPCR anaylsis of *Dmrt1* in XY and XX *Rspo1^KO^Sox9^cKO^* gonads at P0. *Dmrt1* is significantly up-regulated in the XY *Rspo1^KO^Sox9^cKO^* gonads, indicating that ablation of both *Rspo1* and *Sox9* promotes Sertoli cell differentiation. *Dmrt1* is not up-regulated in XX *Rspo1^KO^Sox9^cKO^* gonads. Indeed, at P0 few Sertoli cells have differentiated in XX *Rspo1^KO^Sox9^cKO^* gonads in comparison to XY *Rspo1^KO^Sox9^cKO^* gonads (see Figure 3). In addition it is noteworthy that *Dmrt1* transcription occurs in the XY *Sox9cKO* but the protein is not detected (see same figure panel A). This suggests that the *Rspo1* signaling pathway antagonizes the male pathway, at least partly at the post-transcriptional level, as shown for two other genes *Wnt4* and *Fgf9* [56], [72].
(TIF)

Figure S5 Histological analysis of XY and XX *Rspo1^KO^Sox9^cKO^* gonads at P0, P12 and P21. Differentiation of seminiferous tubules containing germ cells in XY *Rspo1^KO^ Sox9^cKO^*hypoplasic testis (c, h, m) and XY controls (a, f, k). In XX *Rspo1^KO^Sox9^cKO^* (d, i, n), the gonads develop as ovotestes containing both seminiferous tubules and follicles (white star indicates a section of a follicle and white arrowhead shows a section (n) of a seminiferous tubule). However, as previously shown [73], the XX germ cells did not survive in post-natal seminiferous cords. In contrast, germ cells survived until P12 and some until P21 in the XY *Rspo1^KO^Sox9^cKO^* seminiferous tubules (h and m respectively) (scale bar: 50 µm). Controls XY (a, f, k) and XX (e, j, o), XY *Sox9^cKO^* (b, g, I) and XY *Rspo1^KO^Sox9^cKO^* (c, h, m), XX *Rspo1^KO^Sox9^cKO^* (d, i, n).
(TIF)

Figure S6 Mixed germ cell differentiation in XY *Rspo1^KO^Sox9^cKO^* gonads at 14.5 d*pc*. *In situ* hybridization using a riboprobe for *Sox9* (a–d), *Cyp26b1* (e–h), *Stra8* (i–l) and *Oct4* transcripts (m–p) shows lack of *Sox9* expression in the XY *Sox9^cKO^* (b) XY *Rspo1^KO^Sox9^cKO^* (c) and the XX control (d) gonads. Germ cells in XY *Sox9^cKO^* (j) and XX (i) control gonads have entered meiosis in as evidenced by robust *Stra8* expression and weak expression of primordial germ cell marker *Oct4* (n and p respectively). XY *Rspo1^KO^Sox9^cKO^* mutants (k) showed *Stra8* expression at the periphery of the E14.5 gonad indicating these few cells have undergone meiosis (k) while the remaining germ cells were quiescent, thus, express *Oct4* (o). Few *Cyp26b1* expressing cells were detected in XY *Rspo1^KO^Sox9^cKO^* gonads (g). G: gonad, K: Kidney. XY (a, e, i, m) and XX (d, h, l, p) *Rspo1* ^+/−^; *Sox9 ^flox/flox^* controls, XY *Sox9^cKO^* (b, f, j, n) and XY *Rspo1^KO^Sox9^cKO^* (c, g, k, o).
(TIF)
