# Supplementary figures and images for: Testicular Differentiation Occurs in Absence of R-spondin1 and Sox9 in Mouse Sex Reversals
Source: PLoS Genet. 2012 Dec 27;8(12):e1003170. doi: 10.1371/journal.pgen.1003170 (PMC3531470; doi:10.1371/journal.pgen.1003170)

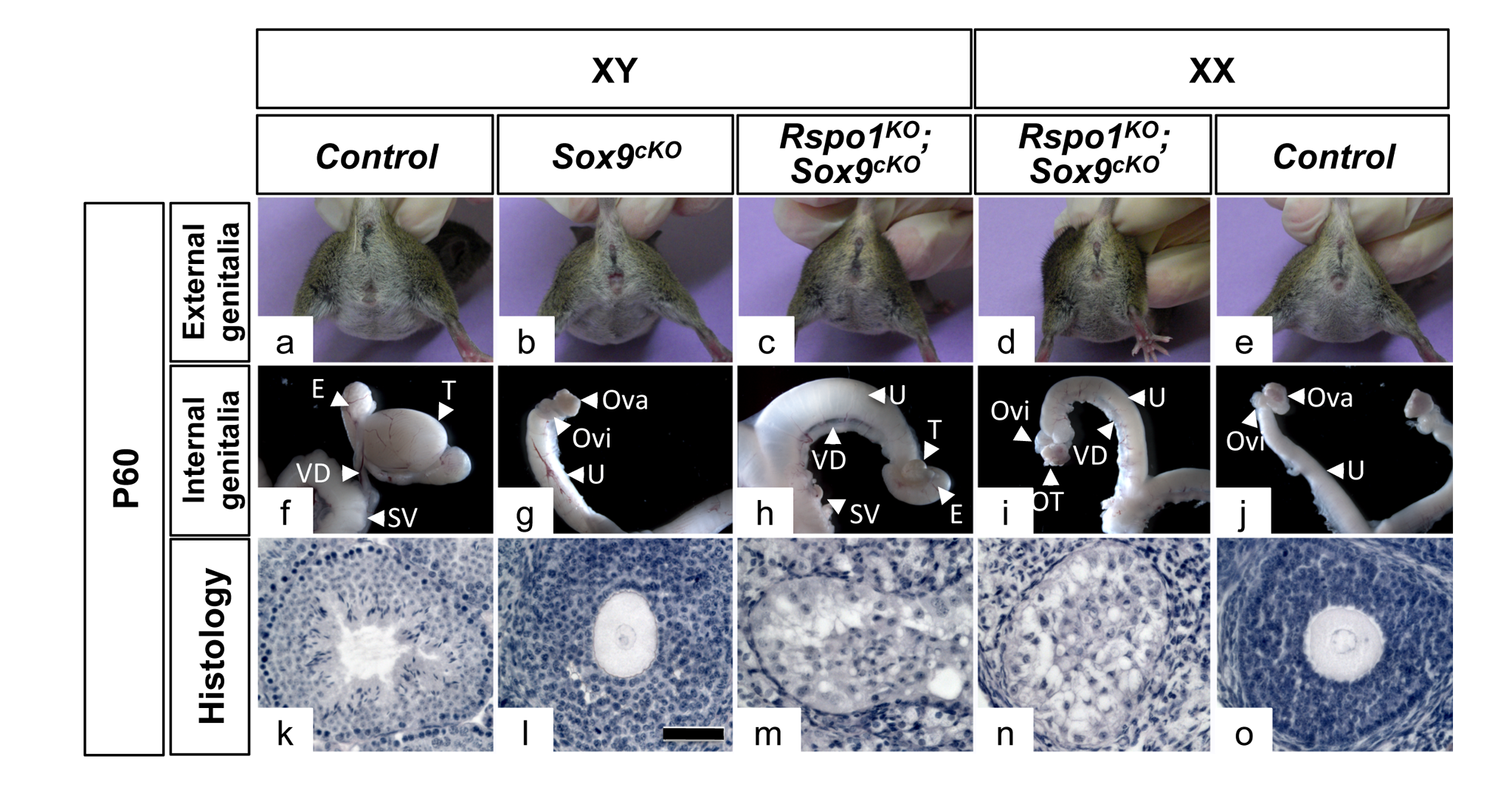

Supplement: Figure S1 — Testicular differentiation in XY and XX Rspo1KOSox9cKO mice. External genitalia of XX control mice (e) is similar to XY Sox9cKO (b), XY and XX Rspo1KOSox9cKO mice (c and d respectively) at 2 months of age. The internal genitalia of XY and XX Rspo1KOSox9cKO mice (h, i) show epididymides (E), vasa deferentia (VD) and seminal vesicles (SV), as in XY males (f) but also uterine horns (U) and oviducts (Ovi) as in XX controls (j) or XY Sox9cKOmice (g). PAS stained histological sections of XY and XX Rspo1KOSox9cKO gonads (m, n) show seminiferous tubules lacking germ cells because hypoplasia of germ cells occurred in these tubules. XY Sox9cKO gonads (l) are similar to ovaries (o) (scale bar, 50 µm). XY (a, f, k) and XX (e, j, o) Rspo1+/−;Sox9flox/floxcontrols, XY Sox9cKO gonads (b, g, l), XY (c, h, m) and XX (d, i, n) Rspo1KOSox9cKO gonads respectively. (TIF) [file pgen.1003170.s001.tif]

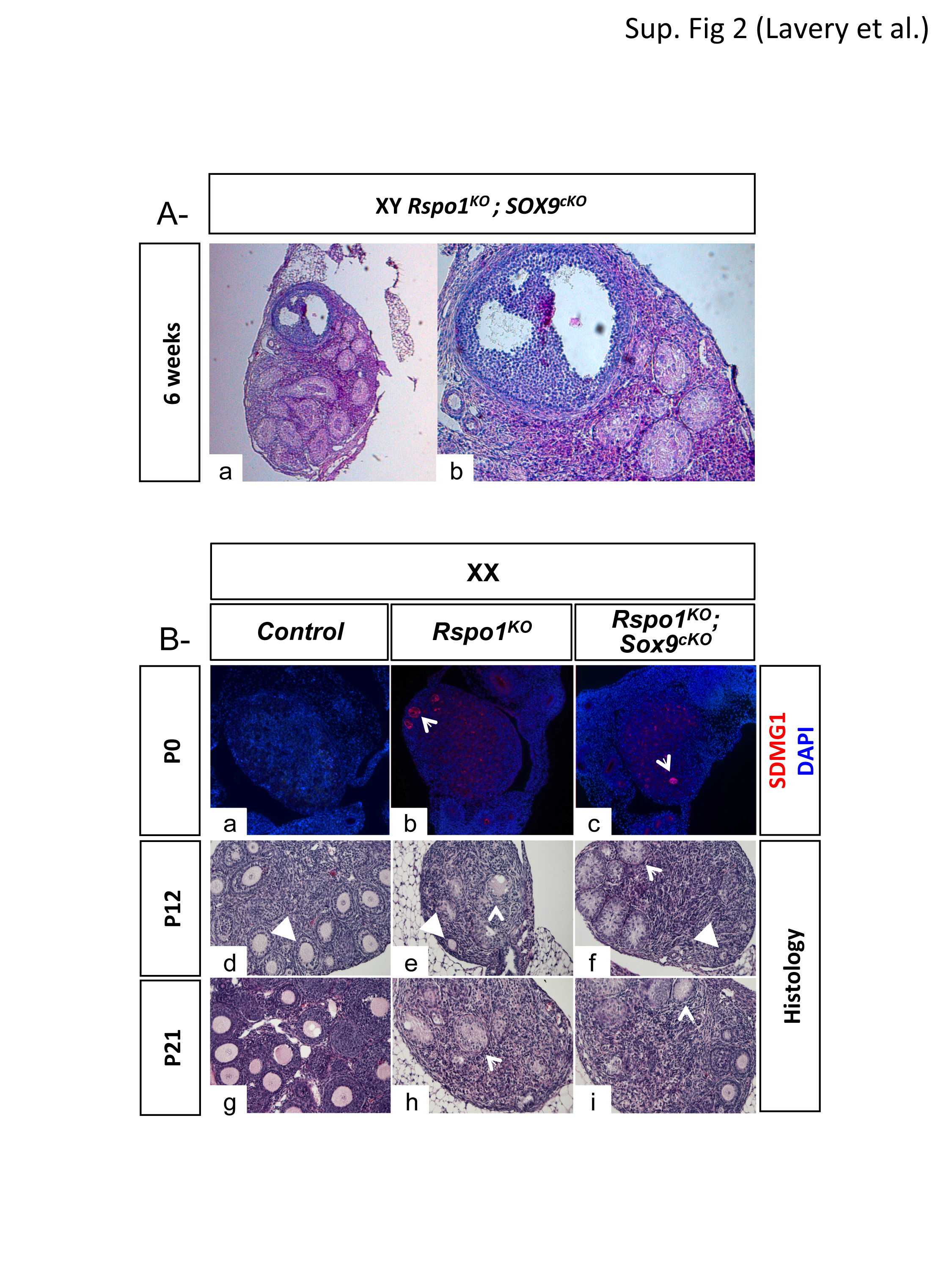

Supplement: Figure S2 — A- XY Rspo1KO Sox9cKO gonad containing a single follicle. XY Rspo1KO Sox9cKO gonad with a single grossly large follicle was located near the entrance of the oviduct (a). This follicle contained three oocytes and was observed in XY Rspo1KO Sox9cKO gonads on rare occasions (b). B- Comparison of XX Rspo1KOand XX Rspo1KO Sox9cKO gonads. Immunofluorescence detection of SDMG1 in Sertoli cells (cytoplasmic) of XX Rspo1KO (b) and XX Rspo1KO Sox9cKO (c) gonads at P0. Some sex cords are clearly visible in XX Rspo1KO and XX Rspo1KOSox9cKO in contrast to XX control gonads (a). Histological analysis of XX Rspo1KO and XX Rspo1KO Sox9cKO gonads at P12 and P21 (e, h and f, i respectively) show the presence of seminiferous tubules and follicles in comparison to XX controls containing only follicles (d, g). Empty and filled arrowheads indicate testis cords and follicles, respectively. (TIF) [file pgen.1003170.s002.tif]

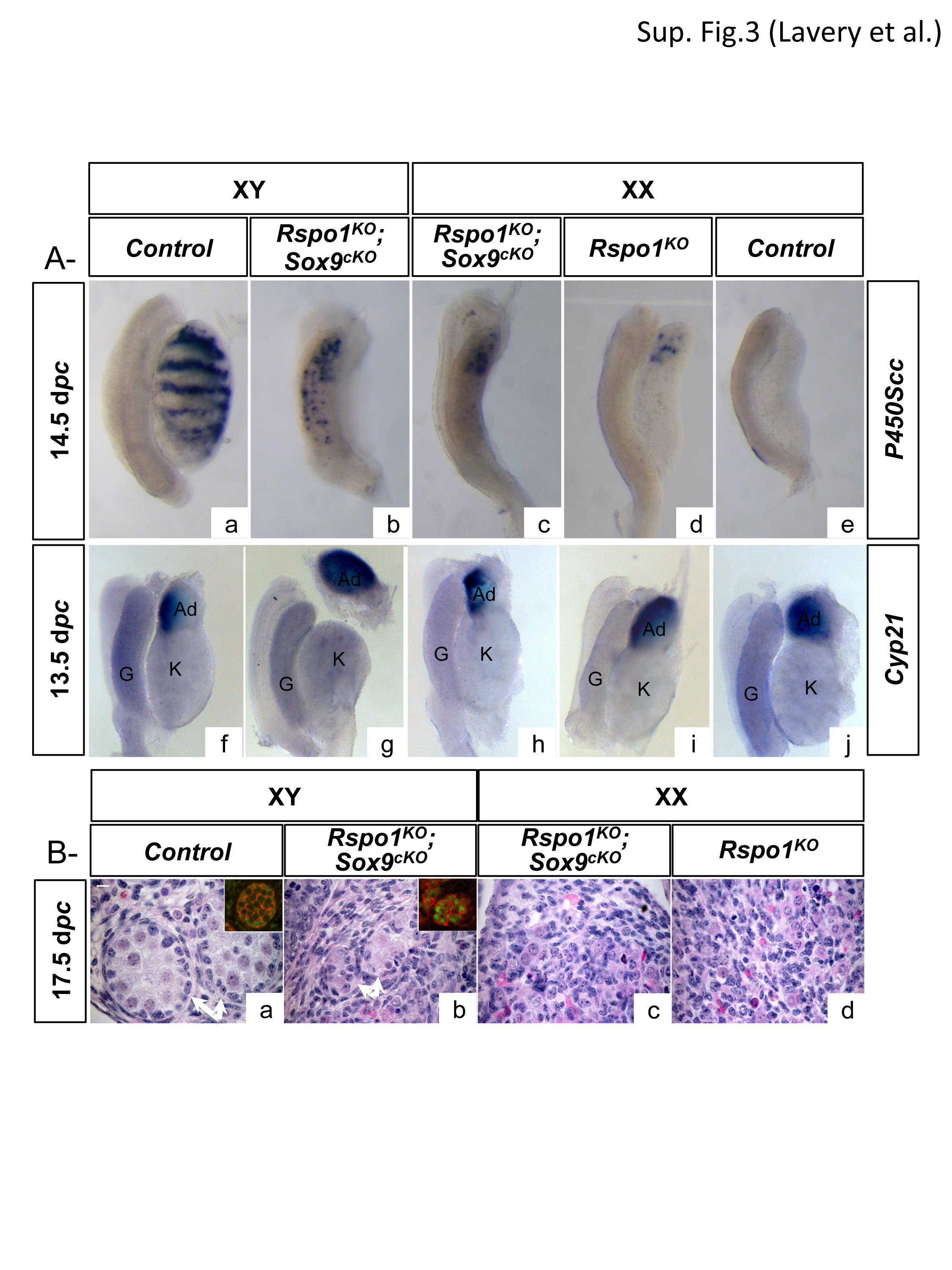

Supplement: Figure S3 — A-Expression of the steroidogenic marker P450Scc and Cyp21 in XY Rspo1KOSox9cKO gonads. Whole-mount in situ hydridization of gonads at 14.5 dpc and 13.5 dpc. P450Scc is expressed in XY Rspo1KOSox9cKO gonads (b), and in XY controls (a) but not in XX controls (e). P450Scc, was expressed in cells at the anterior part of the XX Rspo1KOSox9cKO and XX Rspo1KO gonads (c and d respectively) at 14.5 dpc. Cyp21 was strongly expressed in the adrenals (f, g, h, i, j), whereas no signal was detected in the gonads at 13.5 dpc (f, g, h, i, j). Ad: adrenal, G: gonad, K: kidney. B-Delayed testicular cords formation in XY and XX Rspo1KOSox9cKO gonads. Haematoxylin and eosin stained histological sections of XY and XX Rspo1KOSox9cKO gonads at 17.5 dpc show that some sex cords are forming in the XY Rspo1KOSox9cKO (b) gonads in contrast to the XY controls (a) containing already formed sex cords. In the littermates XX Rspo1KOSox9cKO and Rspo1KO gonads (c and d respectively), no sex cords were observed at this stage (scale bar, 10 µm). Insets in a and b show AMH (Red) and DMRT1 (green), two markers of Sertoli cells highlighting sex cords at 16.5 dpc. Sex cords were rare in XY Rspo1KOSox9cKO gonads and absent from XX Rspo1KOSox9cKO gonads at this stage. (TIF) [file pgen.1003170.s003.tif]

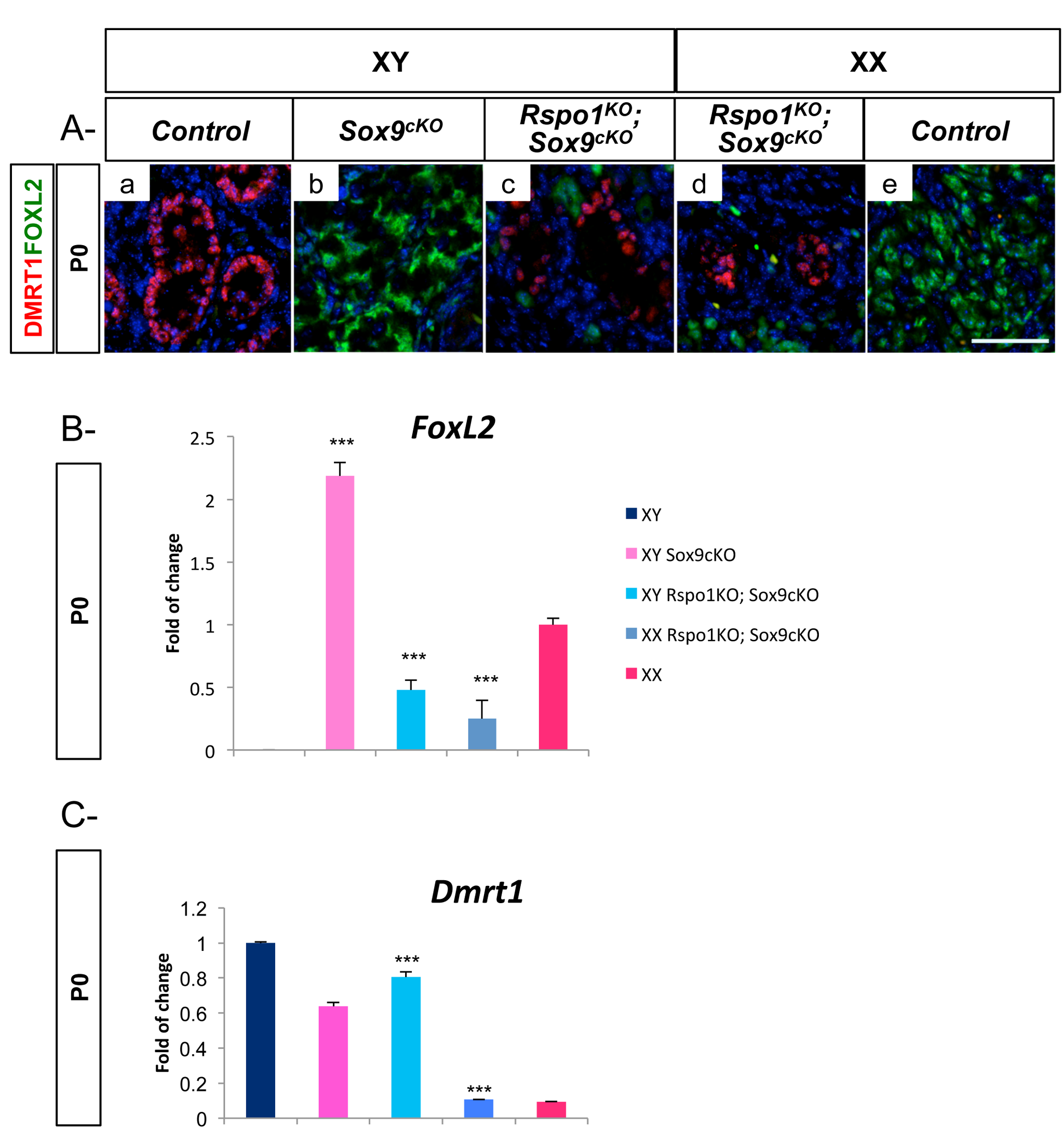

Supplement: Figure S4 — Detection of DMRT1 and FOXL2 in XY and XX Rspo1KOSox9cKO gonads at P0. A- Immunofluorescence analysis of DMRT1 and FOXL2 in XY and XX Rspo1KOSox9cKO gonads at P0. DMRT1 (red), a marker of postnatal Sertoli cells, was detected in the XY control (a), XY Rspo1KOSox9cKO and XX Rspo1KOSox9cKO gonads (c and d respectively) while in XY Sox9cKO (b) and XX (e) control gonads DMRT1 was not detected. The ovarian protein FOXL2 (green) was detected in XY Sox9cKO and XX control gonads, and few FOXL2-positive cells (granulosa cells) were found within the XY Rspo1KOSox9cKO (c) and XX Rspo1KOSox9cKO (d) gonads. B- qPCR anaylsis of Foxl2 in XY and XX Rspo1KOSox9cKO gonads at P0. Foxl2 is significantly up-regulated in the XY Sox9cKO compared to XY and XX Rspo1KOSox9cKO indicating that ablation of Rspo1 leads to reduced Foxl2 expression at a transcriptional level. C- qPCR anaylsis of Dmrt1 in XY and XX Rspo1KOSox9cKO gonads at P0. Dmrt1 is significantly up-regulated in the XY Rspo1KOSox9cKO gonads, indicating that ablation of both Rspo1 and Sox9 promotes Sertoli cell differentiation. Dmrt1 is not up-regulated in XX Rspo1KOSox9cKO gonads. Indeed, at P0 few Sertoli cells have differentiated in XX Rspo1KOSox9cKO gonads in comparison to XY Rspo1KOSox9cKO gonads (see Figure 3). In addition it is noteworthy that Dmrt1 transcription occurs in the XY Sox9cKO but the protein is not detected (see same figure panel A). This suggests that the Rspo1 signaling pathway antagonizes the male pathway, at least partly at the post-transcriptional level, as shown for two other genes Wnt4 and Fgf9 [56], [72]. (TIF) [file pgen.1003170.s004.tif]

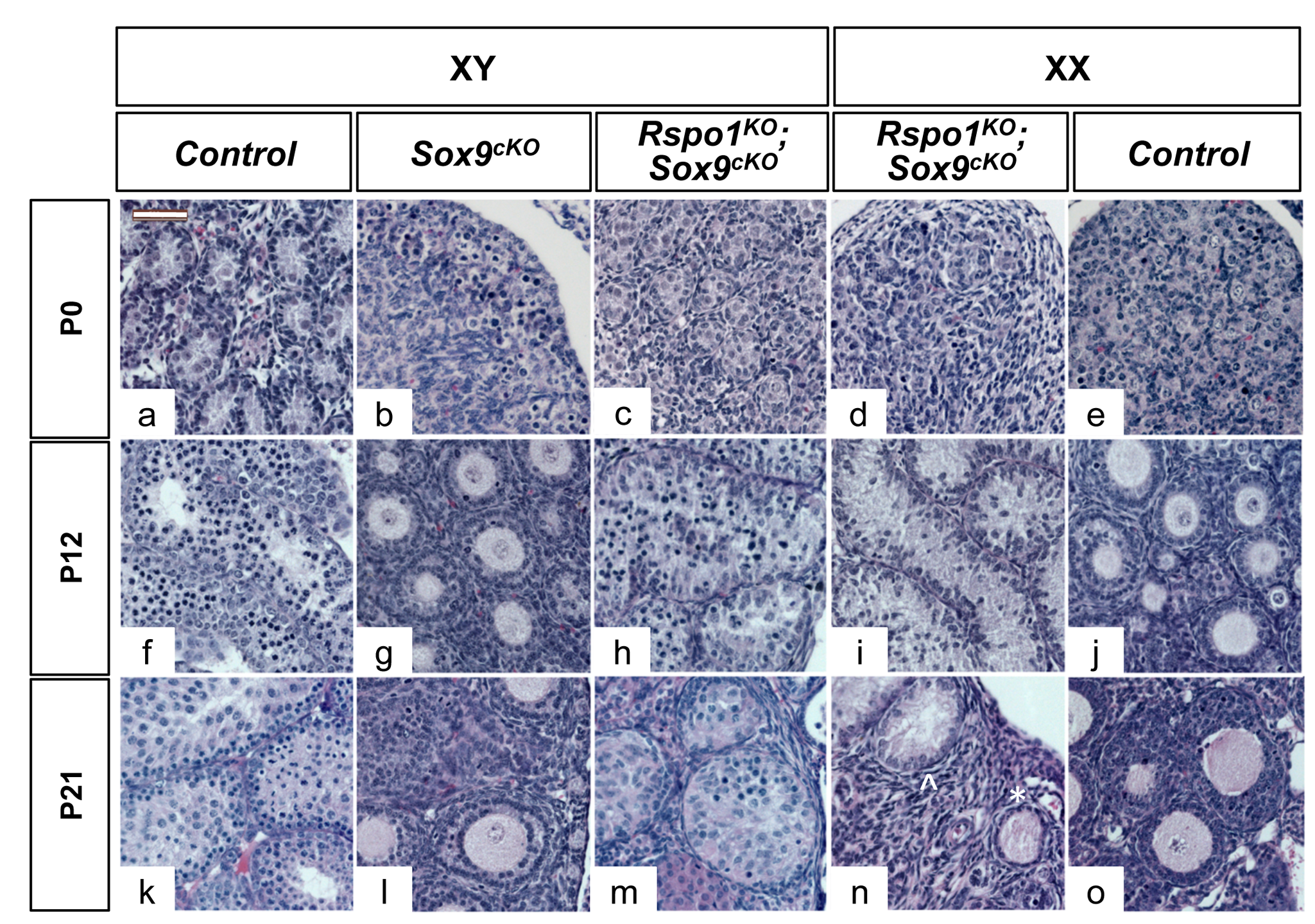

Supplement: Figure S5 — Histological analysis of XY and XX Rspo1KOSox9cKO gonads at P0, P12 and P21. Differentiation of seminiferous tubules containing germ cells in XY Rspo1KO Sox9cKOhypoplasic testis (c, h, m) and XY controls (a, f, k). In XX Rspo1KOSox9cKO (d, i, n), the gonads develop as ovotestes containing both seminiferous tubules and follicles (white star indicates a section of a follicle and white arrowhead shows a section (n) of a seminiferous tubule). However, as previously shown [73], the XX germ cells did not survive in post-natal seminiferous cords. In contrast, germ cells survived until P12 and some until P21 in the XY Rspo1KOSox9cKO seminiferous tubules (h and m respectively) (scale bar: 50 µm). Controls XY (a, f, k) and XX (e, j, o), XY Sox9cKO (b, g, I) and XY Rspo1KOSox9cKO (c, h, m), XX Rspo1KOSox9cKO (d, i, n). (TIF) [file pgen.1003170.s005.tif]

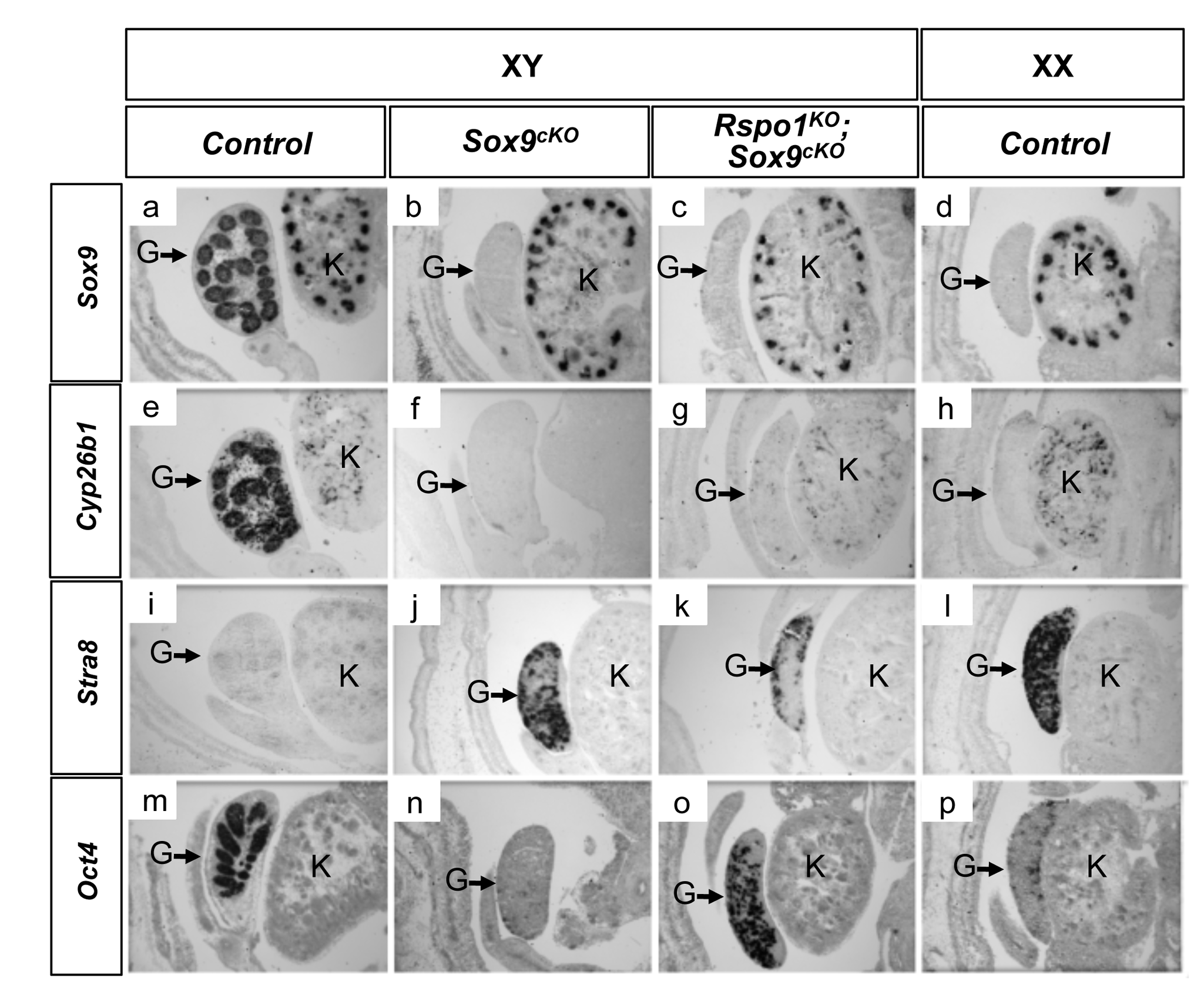

Supplement: Figure S6 — Mixed germ cell differentiation in XY Rspo1KOSox9cKO gonads at 14.5 dpc. In situ hybridization using a riboprobe for Sox9 (a–d), Cyp26b1 (e–h), Stra8 (i–l) and Oct4 transcripts (m–p) shows lack of Sox9 expression in the XY Sox9cKO (b) XY Rspo1KOSox9cKO (c) and the XX control (d) gonads. Germ cells in XY Sox9cKO (j) and XX (i) control gonads have entered meiosis in as evidenced by robust Stra8 expression and weak expression of primordial germ cell marker Oct4 (n and p respectively). XY Rspo1KOSox9cKO mutants (k) showed Stra8 expression at the periphery of the E14.5 gonad indicating these few cells have undergone meiosis (k) while the remaining germ cells were quiescent, thus, express Oct4 (o). Few Cyp26b1 expressing cells were detected in XY Rspo1KOSox9cKO gonads (g). G: gonad, K: Kidney. XY (a, e, i, m) and XX (d, h, l, p) Rspo1 +/−; Sox9 flox/flox controls, XY Sox9cKO (b, f, j, n) and XY Rspo1KOSox9cKO (c, g, k, o). (TIF) [file pgen.1003170.s006.tif]
